# Seasonal differences in brown adipose tissue density and pulse rate variability in a thermoneutral environment

**DOI:** 10.1186/s40101-018-0166-x

**Published:** 2018-02-21

**Authors:** Shinsuke Nirengi, Naoki Sakane, Shiho Amagasa, Sawako Wakui, Toshiyuki Homma, Yuko Kurosawa, Takafumi Hamaoka

**Affiliations:** 1 Division of Preventive Medicine, Clinical Research Institute, National Hospital Organization Kyoto Medical Center, 1-1 Mukaihata-cho, Fukakusa, Kyoto, 612-8555 Japan; 2 Department of Preventive Medicine and Public Health, Tokyo Medical University, 6-1-1 Shinjuku, Shinjuku-ku, Tokyo, 160-8402 Japan; 3 Faculty of Health and Sports Science, Juntendo University, 1-1 Hiragagakuendai, Inzai, Chiba, 270-1695 Japan; 4 Faculty of Sports and Health Science, Daito Bunka University, 560 Iwadono, Higashimatsuyama-shi, Saitama, 355-8501 Japan; 5 Department of Sports Medicine for Health Promotion, Tokyo Medical University, 6-1-1 Shinjuku, Shinjuku-ku, Tokyo, 160-8402 Japan

**Keywords:** Near-infrared time-resolved spectroscopy, Brown adipose tissue, Seasonality, Autonomic nervous system

## Abstract

**Background:**

Brown adipose tissue (BAT) is sympathetically activated and induces thermogenesis during cold exposure, thereby influencing energy expenditure and body fat levels. The very low frequency (VLF) components of pulse rate variability could be a form of thermogenic sympathetic nervous activity, but no clear relationship has yet been reported between VLF activity and BAT density. We therefore aimed to evaluate the association between them.

**Methods:**

We enrolled 20 adults in winter and 20 matched adults in summer. We assessed BAT densities based on total hemoglobin concentrations ([total-Hb]) measured with near-infrared time-resolved spectroscopy. We calculated VLF activity from pulse rate variability measurements.

**Results:**

BAT density ([total-Hb]; winter 70.5 ± 17.0 μM, summer 57.8 ± 18.3 μM) and VLF activity (winter 6.7 ± 0.8, summer 6.1 ± 0.9) were significantly higher in winter than in summer (*P* < 0.05). However, there was no significant correlation between VLF activity and BAT density in either season.

**Conclusion:**

Each parameter exhibited seasonal variation, but we failed to observe any significant correlations.

## Background

The obesity pandemic necessitates effective therapies to prevent consequent diseases. In mammals, adipose tissue is found in two forms: white adipose tissue and brown adipose tissue (BAT) [1]. White adipose tissue stores energy in the form of triacylglycerol, whereas BAT is important for thermogenesis and homeostasis in small mammals. BAT activation in mice promotes energy expenditure, reduces adiposity, and protects against diet-induced obesity [2]. BAT activation is therefore being investigated as an anti-obesity tool.

Functional BAT was recently detected in healthy adults using ^18^F-fluorodeoxyglucose positron emission tomography combined with computed tomography (^18^FDG-PET/CT) [3–6]. Intriguingly, BAT is more often observed in lean individuals than in obese ones [3], as is the case in animals [1]. The BAT provides non-shivering thermogenesis through sympathetic nervous system (SNS) activity in animals [1]. BAT activity and mass increase in winter [3–7], and they may be related to SNS activity in humans. Indeed, β_3_-adrenergic agonists [8] and consumption of foods [9] that promote SNS activity [10] increase BAT activity and mass in humans, but no reports have yet identified a relationship between BAT mass and SNS activity in humans. Near-infrared time-resolved spectroscopy (NIR_TRS_) is a method for evaluating BAT density that is simpler and less invasive than ^18^FDG-PET/CT [9, 11, 12]. NIR_TRS_ permits evaluations of BAT vascularity, which can be used to assess BAT density. NIR_TRS_ thus permits direct quantification of BAT rather than of physiological reactions. Indeed, our previous study [11] showed that BAT activity and mass, as evaluated with ^18^FDG-PET/CT, are related to BAT density, as evaluated with NIR_TRS_ (*r* = 0.73), under both thermoneutral and cold conditions. Furthermore, our longitudinal study [9] showed that the total hemoglobin concentration ([total-Hb]) positively correlates with BAT activity, as measured with ^18^FDG-PET/CT, during repeated thermogenic capsiate intake, which is known to increase BAT activity and mass in both sexes. NIR_TRS_ therefore permits BAT density evaluations without cold exposure.

Pulse rate variability (PRV) measurements are used in clinical practice and research to noninvasively estimate autonomic nervous system (ANS) function [13, 14] and heart rate variability [15]. The PRV frequency domain method can distinguish high-frequency (HF > 25 Hz) components that purely reflect parasympathetic nervous system (PNS) activity from low-frequency (LF < 0.15 Hz) and very low frequency (VLF 0.003–0.15 Hz) components that reflect SNS [16] and PNS activities [17]. A previous study [18] reported that SNS activity is elevated in winter. Parts of the VLF band much lower than 0.1 Hz may reflect thermoregulatory control [19, 20], so the VLF activation observed in response to thermogenic stimuli implies that VLF-associated activity reflects thermogenic regulation, which in turn reflects regulation of energy metabolism primarily performed by the SNS [16, 21, 22]. In contrast, a study that used pharmacological blockade methods [17] suggested that the VLF band predominantly reflects parasympathetic contributions.

Because BAT is thermogenic and is regulated solely by the SNS, its activity may be related to VLF activity. However, no studies have investigated the relationship between BAT density and VLF activity. We therefore aimed to compare SNS activity, especially VLF-reflected SNS activity, in winter and summer using the PRV frequency domain method and to test the relationship between BAT density and VLF activity.

## Methods

This study was conducted in the summer and winter of 2014 in Shiga Prefecture, Japan. The study protocols were approved by Ritsumeikan University’s Institutional Review Board (approval number: 2013-036) and performed in accordance with the principles of the Declaration of Helsinki (Fortaleza 2013). Written informed consent was obtained from all participants.

### Subjects

**Fig. 1:**
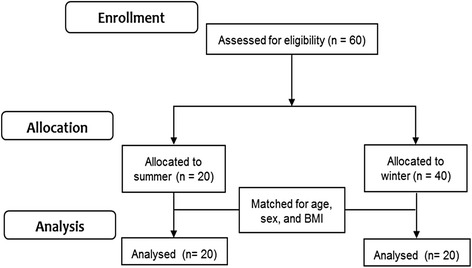
Recruitment and follow-up diagram

We recruited university students with poster advertisements and emails to the student body. The volunteers included 42 men and 18 women. After excluding habitual smokers, heavy drinkers (> 30 g of ethanol per day), and subjects who took medications, we assigned 20 students each to the summer and winter groups. These two groups were matched in age, sex, and body mass index (BMI) (Fig. 1).

The subjects were instructed to avoid high-intensity physical activity on the days before and of the test. The women were measured during their luteal phases as predicted from their menstrual cycles and last menstruations.

### Anthropometric and blood pressure parameter measurements

We evaluated the body mass, fat mass, percent body fat, lean body mass, and bone mass of each subject with a Lunar Prodigy dual-energy X-ray absorptiometer (GE Healthcare, Chicago, IL). After allowing the subjects to rest for 10 min, we measured their systolic blood pressure, diastolic blood pressure, and heart rate using an HBP-9020 automated sphygmomanometer (Omron, Kyoto, Japan).

### BAT density measurements

We determined BAT density in the supraclavicular region by measuring the [total-Hb] using a TRS-20 NIR_TRS_ device (Hamamatsu Photonics, Hamamatsu, Japan) for 5 min at 27 °C as previously described [9, 11, 12]. The distance between the emitter and detector was set at 30 mm [23].

The tissue was illuminated with 100-ps pulses of 760-, 800-, and 830-nm light from a 200-μm core diameter optical fiber with a 100-ps full-width at half-maximum, a 5-MHz repetition rate, and an 80-μW average power at each wavelength. The emitted photons penetrated the tissue and were reflected to a 3-mm diameter optical bundle fiber, through which they were sent to a photomultiplier tube for single-photon detection and a signal processing circuit for time-resolved measurement. Using the non-linear least squares method, the digitized temporal profile data from an in vitro sample or tissue were fitted with a theoretical temporal profile derived from the analytical solution of photon diffusion theory with a semi-infinite homogeneous model in reflectance mode. After convolution with the instrumental response function such that the time response of the instrument itself could be compensated for, the absorption coefficients and reduced scattering coefficients at 760, 800, and 830 nm were obtained. The absolute [total-Hb] values were then determined using a least squares fitting method [19]. The NIR_TRS_ system collected data at a 0.1-Hz sampling rate. The coefficient of variation for repeated [total-Hb] measurements was 4.9% [11].

### ANS parameters

PRV was measured using a sphygmograph (TAS9 Pulse Analyzer Plus, YKC, Tokyo, Japan) for 15 min at 27 °C with subjects in the supine position and wearing light clothing. The frequency domain information was automatically analyzed with a fast Fourier transform [24, 25]. This analysis involved 1000 samples, a 300-ms pulse interval resampling frequency, and a Hanning window function. The correspondence of PRV with heart rate variability in the resting state is well documented [13, 26]. We defined the VLF, LF, HF, and total power (TP) components as the areas under the spectral peaks within the 0.0033–0.04 Hz, 0.04–0.15 Hz, 0.15–0.4 Hz, and 0.0033–0.4 Hz ranges, respectively. We assessed sympathetic activity by calculating the LF to HF ratio (LF/HF).

### Dietary intake and physical activity recordings

Dietary habits during the preceding month were assessed using a validated, brief, self-administered diet history questionnaire that queried the consumption frequencies for 56 foods and beverages and 9 dishes that are commonly consumed in Japan. Daily intakes of energy, proteins, fats, and carbohydrates were calculated from the responses [27]. Daily steps and activity-related energy expenditures were estimated using Health Counter HJ-710IT pedometers (Omron Healthcare Corp., Kyoto, Japan), and weekly means were calculated.

### Statistical analyses

Data are expressed as means ± standard deviations. The ANS parameters were not normally distributed (Shapiro-Wilk test, *P* <  0.05), so they were log-transformed. We initially used Student’s *t* test for between-season comparisons of ANS parameters and then repeated the comparisons for significant differences using analysis of covariance after controlling for age, sex, BMI, and log-TP. We tested for partial correlations between ANS parameters and anthropometric parameters while controlling for age and sex. The analysis of covariance data was expressed as means and 95% confidence intervals (95% CI). We calculated Pearson correlation coefficients for simple correlation analyses. Statistical significance was defined as *P* < 0.05. Pair-matching was performed using Easy R software (Saitama Medical Center, Jichi Medical University, Saitama, Japan) [28], but all other statistical analyses were performed using SPSS version 19 (IBM, Armonk, NY).

## Results

Table 1 shows the anthropometric and blood pressure measurements in each season. There were no significant between-season differences in height, bodyweight, BMI, body fat mass, body fat proportions, bone mass, blood pressure, heart rate, physical activity (step numbers and energy expenditure), or dietary intake of fats, proteins, carbohydrates, or total calories.

**Table 1:** Participant characteristics

Variables	Summer (*n* = 20)	Winter (*n* = 20)	*P* value
Age, years	21.0 ± 1.2	20.6 ± 0.9	0.19
Female, %	40	40	–
Height, cm	165.7 ± 9.1	166.0 ± 6.6	0.90
Body weight, kg	68.3 ± 14.6	64.9 ± 13.3	0.45
BMI, kg/m^2^	24.7 ± 4.0	23.4 ± 3.6	0.28
Body fat, %	26.0 ± 8.5	23.7 ± 6.0	0.33
Fat mass, kg	17.6 ± 9.2	14.2 ± 5.0	0.16
Lean body mass, kg	47.6 ± 7.8	47.2 ± 9.5	0.89
Bone mass, kg	2.9 ± 0.4	2.8 ± 0.5	0.38
SBP, mmHg	118.6 ± 17.6	120.1 ± 10.5	0.74
DBP, mmHg	64.4 ± 14.4	66.4 ± 7.8	0.60
Heart rate, bpm	71.2 ± 12.9	65.1 ± 8.8	0.09
Steps, per day	7060.6 ± 2478.8	7690.1 ± 2027.8	0.39
Energy expenditure, kcal/day	218.5 ± 93.2	193.0 ± 64.2	0.32
Energy intake, kcal/day	1997 ± 718	1787 ± 682	0.35
Protein intake, g/day	63.4 ± 22.7	63.5 ± 25.2	0.99
Fat intake, g/day	58.0 ± 22.5	57.4 ± 19.9	0.93
Carbohydrate intake, g/day	293.7 ± 110.3	239.9 ± 101.6	0.12

Data are reported as means ± standard deviations

*BMI* body mass index, *DBP* diastolic blood pressure, *SBP* systolic blood pressure

The [total-Hb] as an index of BAT density was higher in winter than in summer (70.5 ± 17.0 μM vs. 57.8 ± 18.3 μM, respectively; *P* <  0.01) (Table 2). After controlling for age, sex, BMI, and log-TP, we still observed significant between-season differences in the [total-Hb] (58.2 [95% CI 50.0 to 66.5] μM in winter vs. 70.9 [62.7 to 79.2] μM in summer; *P* <  0.01). Among the ANS parameters, the log-HF PRV components were significantly lower in winter than in summer (6.2 ± 0.7 vs. 7.0 ± 1.2, respectively; *P* <  0.05). Of the SNS parameters, the log-LF PRV components were not significantly different between seasons, but the log-VLF PRV components were significantly higher in winter than in summer (6.7 ± 0.8 vs. 6.1 ± 0.9, respectively; *P* < 0.05), as were the LF/HF ratios (1.1 ± 0.1 vs. 0.9 ± 0.1, respectively; *P* < 0.01) (Table 2). After controlling for age, sex, BMI, and log-TP, we still observed significant between-season differences in the log-VLF PRV components (6.7 [95% CI 6.5 to 7.0] in winter vs. 6.0 [5.8 to 6.2] in summer; *P* < 0.01), log-HF PRV components (6.3 [6.1 to 6.5] in winter vs. 7.0 [6.7 to 7.2] in summer; *P* < 0.05), and log-LF/HF ratios (1.1 [1.0 to 1.1] in winter vs. 0.9 [0.9 to 1.0] in summer; *P* < 0.01). No ANS parameter correlated with daily steps or activity-related energy expenditure. The only significant relationship between ANS parameters and anthropometric parameters after adjusting for age and sex was that between log-VLF and body fat mass (Table 3). After adjusting for age and sex, the [total-Hb] negatively correlated with BMI (*r* = − 0.51, *P* < 0.05), body fat (− 0.65, *P* < 0.05), and fat mass (*r* = − 0.62, *P* < 0.05) but did not significantly correlate with log-TP (*r* = 0.12, *P* = 0.47), log-LF PRV components (*r* = 0.10, *P* = 0.96), log-VLF PRV components (*r* = 0.13, *P* = 0.26), log-HF PRV components (*r* = 0.09, *P* = 0.57), or log-LF/HF ratios (*r* = 0.02, *P* = 0.44). These relationships were not observed in either summer or winter alone. There was therefore no significant relationship between BAT density and any ANS index (Table 3).

**Table 2:** BAT density and autonomic parameters in summer and winter

Variables	Summer (*n* = 20)	Winter (*n* = 20)	*P* value
BAT density, μM	57.8 ± 18.3	70.5 ± 17.0	< 0.05
Autonomic parameters			
Log-TP	7.8 ± 1.0	7.7 ± 0.6	0.63
Log-LF	6.5 ± 1.0	6.5 ± 0.7	0.83
Log-VLF	6.1 ± 0.9	6.7 ± 0.8	< 0.05
Log-HF	7.0 ± 1.2	6.2 ± 0.7	< 0.05
Log-LF/HF	0.9 ± 0.1	1.1 ± 0.1	< 0.01

Data are reported as means ± standard deviations

BAT density was evaluated from total hemoglobin concentrations measured with near-infrared time resolved spectroscopy in the supraclavicular region

*BAT* brown adipose tissue; *HF* high-frequency; *LF* low-frequency; *LF/HF* LF to HF; *TP* total power; *VLF* very low frequency

**Table 3:** Relationship between anthropometric parameters and heart rate variability components representative of the autonomic nervous system

	BMI	% Body fat	Fat mass	Lean body mass	Bone mass	BAT density
Log-TP	− 0.12	− 0.25	− 0.30	− 0.02	− 0.07	0.12
Log-LF	0.00	− 0.20	− 0.21	0.07	− 0.07	0.11
Log-VLF	− 0.24	− 0.22	− 0.31	− 0.17	− 0.24	0.13
Log-HF	− 0.07	− 0.21	− 0.24	0.03	0.07	0.11
LF/HF	0.04	0.04	0.07	0.03	− 0.14	− 0.04

BAT density was evaluated by examining total hemoglobin concentrations with near-infrared time resolved spectroscopy in the supraclavicular region. Partial correlation analyses were conducted with adjustments for age and sex

*BAT* brown adipose tissue, *HF* high frequency; *LF* low frequency, *LF/HF* LF to HF, *TP* total power, *VLF* very low frequency

## Discussion

Our objective was to evaluate seasonal differences in PRV components reflective of ANS activity, and we succeeded in obtaining the first evidence that the VLF PRV components are greater in winter than in summer even after controlling for confounders. Further, the LF/HF ratio, which reflects SNS activity, and the HF PRV components, which reflect PNS activity, were higher and lower, respectively, in winter than in summer.

ANS measurements are widely used for predictions and diagnoses in both clinical practice and laboratory studies [15]. ANS studies have normally been conducted in environments well controlled for factors such as room temperature, but they have not often considered seasonal differences. In many countries, winter is associated with elevated mortality and hospitalization rates, especially from cardiovascular diseases [29, 30], and it is speculated that this arises from environmental temperature differences [31–33]. Many studies have clearly shown the role of the ANS in cardiovascular pathophysiology [34, 35]. This lends plausibility to our observation of seasonal variations in ANS activity. A few other studies have reported seasonal variations in sympathetic activity [18, 36], such as the observation that winter is associated with elevated resting muscle sympathetic nerve burst rates [18] and plasma norepinephrine concentrations [36] and lower variability in coupling intervals between normal beats [37]. Our results are consistent with those of these studies and provide new information about the VLF PRV components. We think that VLF PRV primarily reflects sympathetic thermogenic activities. Previous studies showed that thermogenic perturbations such as acute cold exposure or consumption of spicy foods containing capsaicin selectively increased VLF activity in persons of normal weight but not in persons with obesity [16, 21, 22]. Furthermore, we observed that VLF activity was increased in winter, but that HF activity was decreased in winter. However, we cannot rule out the possibility that PNS activity contributes to VLF activity because a previous study showed that pharmacologically blockading parasympathetic signals attenuated VLF activity [17].

Studies evaluating BAT activity with ^18^FDG-PET/CT report that it increases in winter [3, 7] and that its mechanism might be related to SNS activity. This is consistent with our observation of elevated [total-Hb], an index of BAT density, in winter. However, we observed no correlation between [total-Hb] and SNS activity, and no other PRV study has observed a correlation between BAT density and SNS activity in humans either. This is inconsistent with studies of other SNS-related parameters. BAT activity is increased by β3-adrenergic agonists [8, 38] and inhibited by β-adrenergic antagonists [39]. ^123^I-metaiodobenzylguanidine imaging, which evaluates sympathetic activity, can visualize BAT activity [40]. Surprisingly, ^18^FDG-PET/CT scans in patients with Horner’s syndrome reveal less BAT activity on the neuropathy-affected side than on the unaffected side [41]. Furthermore, patients with pheochromocytoma who exhibit high plasma total metanephrine levels have greater BAT activity than those who exhibit low total metanephrine levels [42]. These studies suggest that human BAT activity is related to SNS activity, though it may also be related to certain humoral substances that can reportedly increase BAT density, such as myokines [43, 44], thyroid hormones [44], and lipokines [45].

We observed no significant between-season differences in blood pressure, but some large studies observed small between-season differences in systolic and diastolic blood pressure that occur in middle-aged individuals and might increase with age [32, 33, 46]. Our participants may have been too few and too young for us to detect such differences.

Our study has several limitations. First, the two groups were composed of different individuals, and the sample size was relatively small. Therefore, although we conducted sex-matching and adjustments for sex, we could not separately analyze the data of men and women. A thorough understanding and careful implementation of a within-subject design will therefore be necessary in future studies. Second, we did not apply cold stimulation. A previous study reported that energy expenditures do not significantly differ between BAT-negative and BAT-positive subjects at thermoneutral temperatures but are significantly higher in BAT-positive subjects at cold temperatures [47]. Therefore, SNS activity might be greater in subjects with high BAT densities if cold exposure were applied. Third, we did not include subjects with extremely high BAT densities, and the correlation coefficients might have been stronger had we included them. Fourth, the NIR_TRS_ method was confined to measuring 4-cm^3^ volumes and could not permit BAT activity evaluations under cold conditions. Future studies should use ^18^FDG-PET/CT to investigate ANS function and BAT activity during cold exposure in humans.

## Conclusions

In conclusion, we showed that the VLF PRV components and the LF/HF ratios were greater in winter than in summer, concomitant with seasonal changes in BAT density, whereas the ANS-related HF PRV components exhibited the opposite seasonal pattern. However, we found no significant correlations between VLF activity and BAT density.

## Abbreviations

[total-Hb]: Total hemoglobin concentration
^18^FDG-PET/CT: ^18^F-fluorodeoxyglucose positron emission tomography combined with computed tomography
ANS: Autonomic nervous system
BAT: Brown adipose tissue
HF: High frequency
LF: Low frequency
NIR_TRS_: Near-infrared time-resolved spectroscopy
PNS: Parasympathetic nervous system
PRV: Pulse rate variability
SNS: Sympathetic nervous system
VLF: Very low frequency
